# Evaluation of neutral oral contrast agents for assessment of the small bowel at abdominal staging CT

**DOI:** 10.1371/journal.pone.0225160

**Published:** 2019-11-14

**Authors:** Till F. Kaireit, Carolin Huisinga, Matti Peperhove, Frank Wacker, Kristina I. Ringe

**Affiliations:** Department of Diagnostic and Interventional Radiology, Hannover Medical School, Hannover, Germany; Northwestern University Feinberg School of Medicine, UNITED STATES

## Abstract

**Background:**

Although neutral oral contrast agents are widely in use, a consensus regarding a standardized protocol in abdominal staging CT does not exist.

**Purpose:**

To test the null hypothesis that there is no quantitative or qualitative difference between water and mannitol for evaluation of the small bowel at abdominal staging CT.

**Material and methods:**

180 patients prospectively underwent abdominal staging CT with oral administration of either 1 liter mannitol solution (n = 88) or water (n = 92). Intestinal distension was measured in 6 different segments of the small intestine. In addition, two radiologists separately evaluated diagnostic image quality with regards to luminal distension (three-point scale) in each segment and the possibility to rule out a possible underlying pathology. Quantitative and qualitative results were compared (Mann-Whitney test).

**Results:**

Quantitatively, intestinal distension was comparable in all segments (p>0.05), except for the horizontal duodenum (p = 0.019). The mean luminal diameter over all intestinal segments was 19.0 mm (18.1–19.9 mm) for the water group and 18.4 mm (17.5–19.2 mm) for the mannitol group, respectively. Qualitatively, ratings were comparable for the first three segments, while distal segments were rated better using mannitol. Side effects were only observed using mannitol (n = 26; 29.5%).

**Conclusions:**

Orally administered water and mannitol solution for evaluation of the small bowel at abdominal staging CT in clinical routine resulted in comparable results for the quantitative, but not for the qualitative analysis. Looking more differentiated at the overall performance, water has advantages in terms of patient comfort, side effects and costs, and can therefore be regarded as noninferior to mannitol in this specific patient group.

## Introduction

Computed tomography (CT) staging examinations have become a cornerstone in diagnosis and follow-up of oncological disease. In case of abdominal imaging, the supplemental administration of a neutral or positive oral contrast agent for distension and assessment of the small intestine has been proven to reduce misinterpretations at CT, both false negative (i.e. tumors and polyps hidden by collapsed bowel loops) as well as false positive (simulation of wall thickening), and thus has been established in clinical routine [1–4].

Recently, the value of positive oral contrast agents has been questioned as the performance of neutral oral contrast agents was demonstrated to be comparable while less inconvenient, e.g. in oncologic staging, emergency settings or more specifically for the evaluation of inflammatory bowel disease [3–8]. Consequently, indications for positive contrast agents are nowadays mostly limited to particular conditions, e.g. to exclude anastomotic insufficiency after small bowel surgery.

Regarding specific protocols for oral administration of neutral contrast agents most experience is gathered from CT studies acquired with the purpose of distinct evaluation of the small bowel. These protocols often involve extensive patient preparation including a low fiber diet for up to three days. In addition, intensive bowel cleaning may be carried out the afternoon before the examination by oral administration of up to two liters of an isotonic non-absorbable electrolyte solution containing polyethylene glycol. For imaging, neutral contrast agents are then administered either orally (i.e. CT enterography) or through a naso-jejunal catheter (i.e. CT enteroclysis) in large amounts (ranging from 1500 to 2500 ml) [9]. A variety of different neutral oral contrast agents are being used including drinking water, oil emulsions or sugar solutions (e.g. mannitol, sorbitol, polyethylene glycol) [10–12], as these additives have been shown to decrease water reabsorption [13–16]. However, such extensive CT protocols are not only uncomfortable for patients, but also time consuming, cost intensive and not practicable in clinical routine with regards to the rising demand of CT examinations especially in an outpatient setting.

Although neutral oral contrast agents are widely in use, a consensus regarding a standardized protocol in abdominal staging CT does not exist. In this setting a dedicated evaluation of the small intestine is not in the focus of interest. To the best of our knowledge, there is no general agreement for the application of neutral contrast agents, including the specific type of contrast agent. The purpose of our study was therefore to test the null hypothesis that there is no quantitative or qualitative difference between non-sparkling mineral water and mannitol solution for evaluation of the small bowel at abdominal staging CT in clinical routine.

## Materials and methods

### Patients

**Fig 1:**
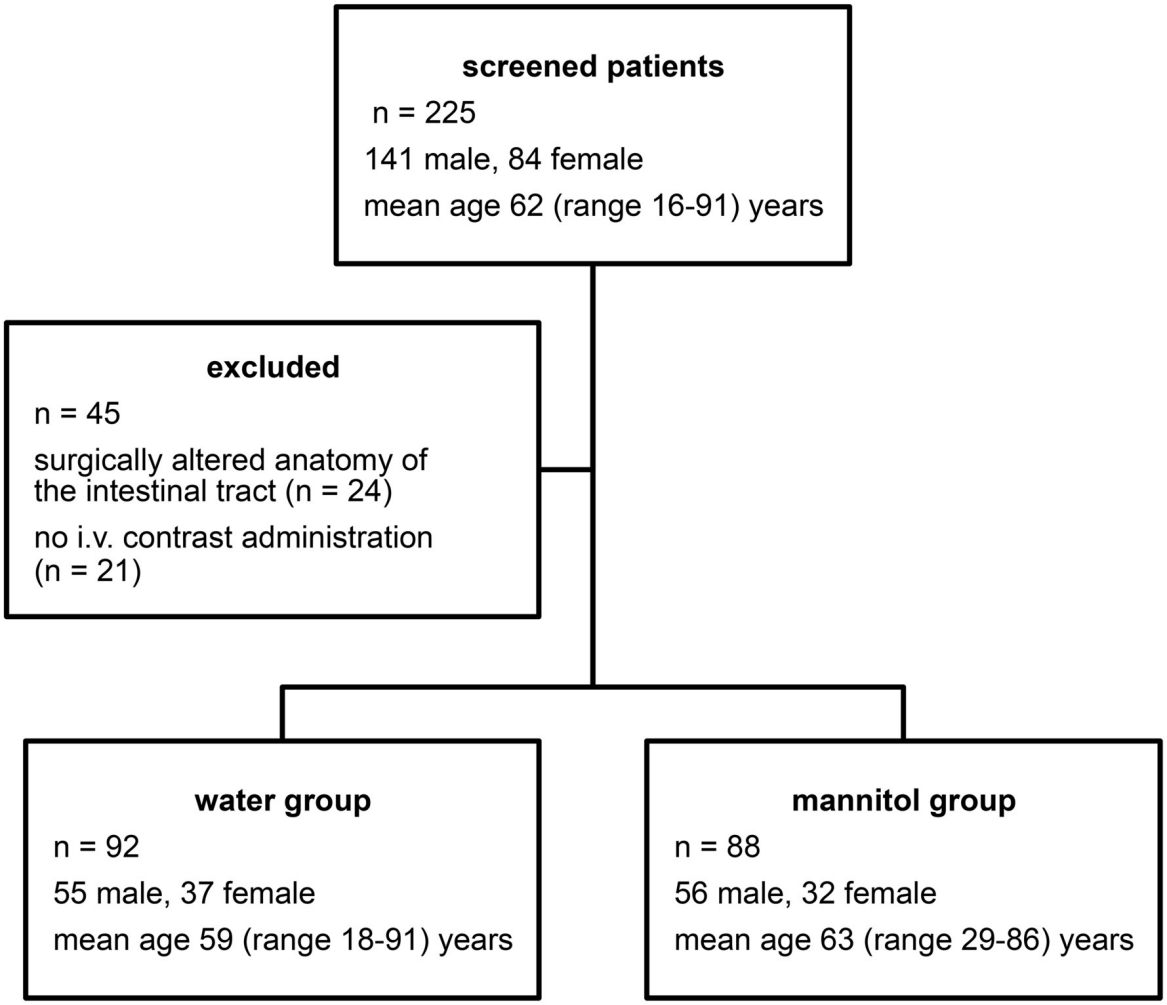
Patient cohort. 225 patients referred for abdominal staging CT were screened for possible study inclusion. Consequently, the final study population consisted of 180 patients.

This prospective observational study was approved by the IRB of Hannover Medical School (Approval Number: 2252–2015). All patients gave written informed consent for study participation. Between March and May 2014, 225 patients referred for staging CT comprising either the abdomen only (including the pelvis) or thorax and abdomen were screened for possible study inclusion. Inclusion criteria were as follows: patient age ≥ 18 years, written informed consent for staging CT, oral administration of either water or mannitol solution. Exclusion criteria were as follows: patient age < 18 years, contraindications for intravenous iodinated contrast agent administration, surgically altered anatomy of the gastrointestinal tract (e.g. status post gastrostomy or ileostomy). 45 patients had to be excluded due to either surgically altered anatomy of the intestinal tract (n = 24) or lack of intravenous contrast administration (n = 21) (Fig 1). Consequently, the final study population consisted of 180 patients (69 female, 111 male; mean age 61 (range 18–91) years).

### CT imaging

**Fig 2:**
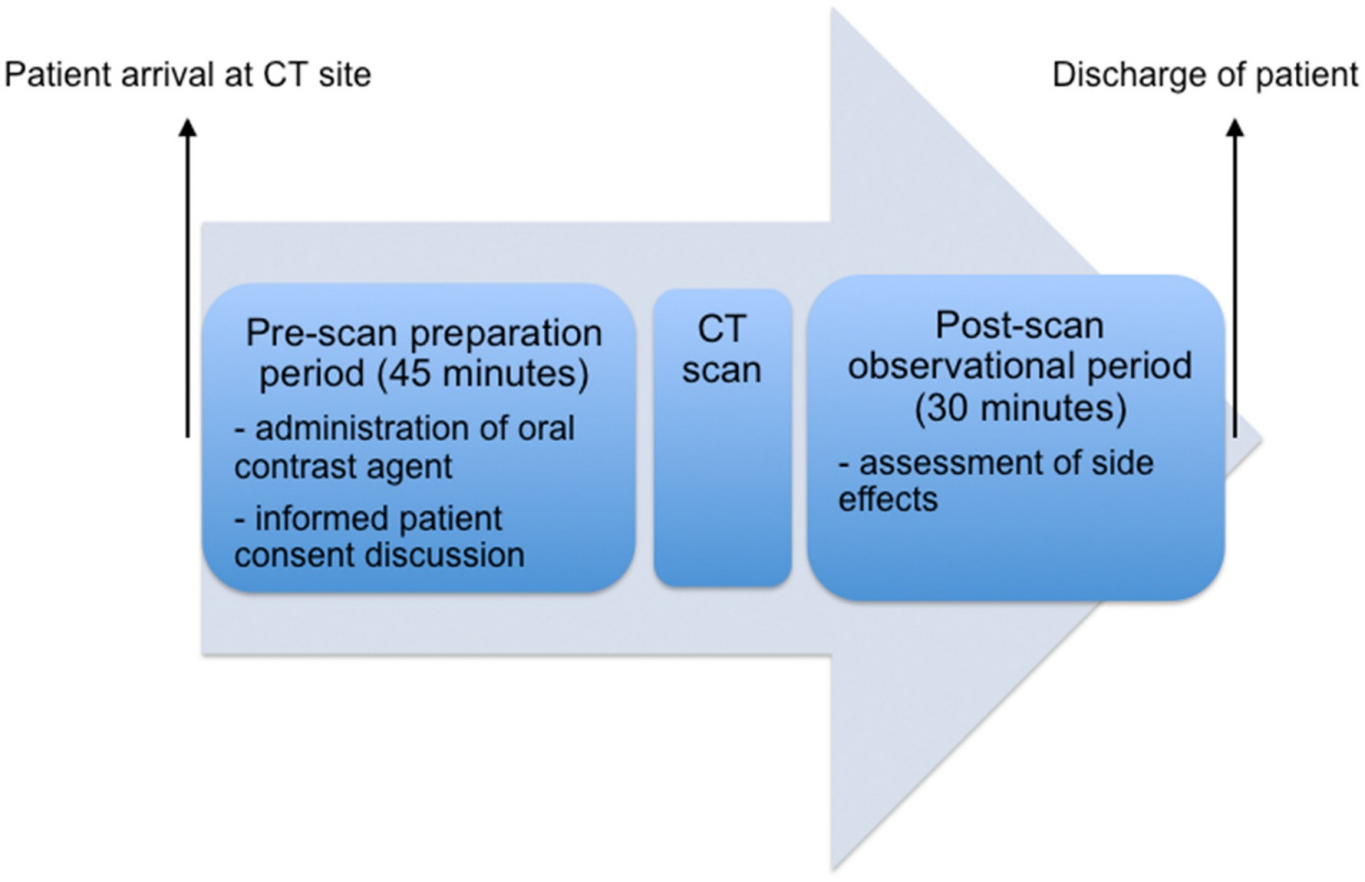
Timeline of the visit of a patient undergoing (thoraco-) abdominal staging CT. After arrival at the CT site, patients received 1 liter of an oral neutral contrast agent and were instructed to drink the full amount within 45 minutes prior to image acquisition. After completion of the CT scan, patients remained in the department over a period of 30 minutes for observational purposes and assessment of potential side effects.

Approximately 45 minutes prior to image acquisition patients consecutively received either 1 liter non-sparkling water (group 1; n = 92) or 1 liter mannitol solution (group 2; n = 88) as a neutral oral contrast agent, both served at room temperature (Fig 2). Allocation of the specific type of oral contrast agent was performed prospectively in dependence on the time period imaging was carried out. Consecutive patients undergoing staging CT between March 15^th^ and April 14^th^ all received water, consecutive patients undergoing staging CT between April 15^th^ and May 16^th^ all received mannitol. Staging CT was performed in the supine position either on a 64-slice scanner (n = 132; Lightspeed VCT, GE HealthCare, Milwaukee, WI, USA) or a 16-slice scanner (n = 48; Lightspeed, GE HealthCare, Milwaukee, WI, USA). Images were acquired in the portal venous phase, after injection of 100 ml of a nonionic iodinated contrast agent (Xenetix^®^ 350, Guerbet, France) followed by a 40 ml saline flush, injected at a flow rate of 4 ml per second. Image acquisition started 15 seconds after bolus detection in the spleen (threshold 250 Hounsfield Units). Scan parameters for image acquisition on the 64-slice scanner were as follows: tube current 120 kV automatic tube modulation, table feed 39.37 mm/ gantry rotation, 1.25 mm slice collimation, 1.0 mm reconstruction interval. Respective parameters for image acquisition on the 16-slice scanner were: tube current 120 kV, automatic tube modulation, table feed 27.5 mm/ gantry rotation, 1.25 mm slice collimation, 1.0 mm reconstruction interval. After completion of the CT scan patients remained in the department over a period of 30 minutes for observational purposes and assessment of potential side effects.

### Image analysis

**Fig 3:**
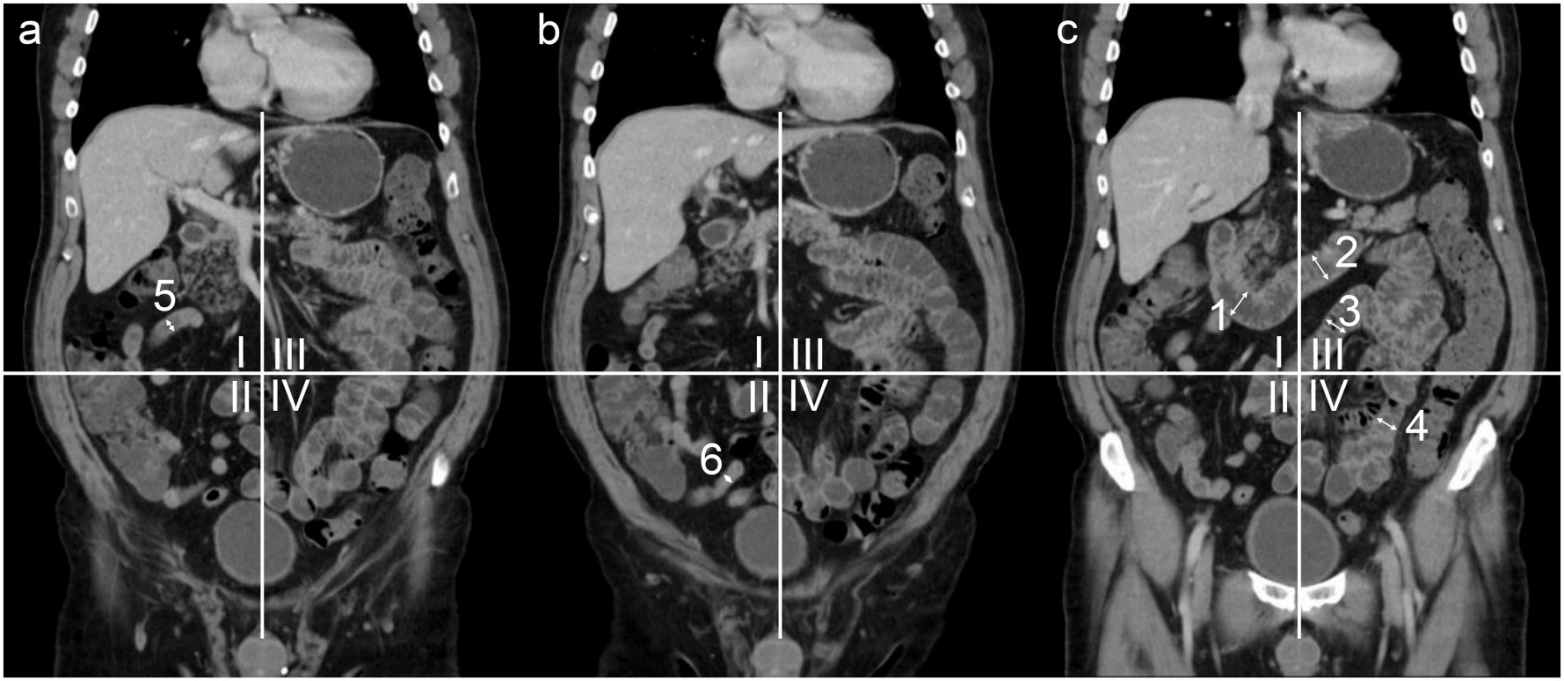
Coronal reformatted CT (three consecutive slices (a-c)) for demonstration of small bowel segment definition. The abdomen is divided into 4 quadrants with the umbilicus as the center point. Representative measurements of the descending (1) and horizontal duodenum (2). Jejunal and ileal segments were defined using the 4-quadrant model: I = upper right quadrant, including the proximal ileum (5); II = lower right quadrant, including the distal ileum (6); III = upper left quadrant, including the proximal jejunum (3); IV = lower left quadrant, including the distal jejunum (4).

Image analysis was performed using a thin client PACS viewer (Visage 7, Pro Medicus, Richmond, Australia). Readouts were performed using sagittal, coronal and transverse reformations in 3 mm average intensity projections. In order to assess the effect of the administered oral contrast agents on the small intestine, the following segments (n = 6) were defined: descending duodenum, horizontal duodenum, proximal jejunum, distal jejunum, proximal ileum and distal ileum. The anatomy of the small intestine was primarily assessed in coronal view. While the duodenal segments can be easily identified, ileal and jejunal loops may be more difficult to distinguish. In order to identify these segments reliably, the abdomen was divided into four quadrants with the umbilicus as the center point, as has been previously suggested by Minordi et al [11]. Small intestine in the left upper quadrant of the abdomen was defined as proximal jejunum, small bowel loops in the left lower quadrant as distal jejunum, loops in the right upper quadrant as proximal ileum and loops in the right lower quadrant as distal ileum [9,11], respectively (Fig 3).

### Quantitative analysis

For quantitative analysis, the luminal distension of each bowel segment at one representative location was measured by two radiologists in consensus (one radiology resident with two years and one board certified radiologist with eleven years of experience in abdominal imaging, respectively). Since the intestinal wall could not always be distinguished from the lumen, the total diameter of the bowel was measured. Both radiologists were blinded to the administered oral contrast agent.

### Qualitative analysis

Qualitative image analysis was performed by two different readers (radiology residents with three and five years of experience in abdominal imaging, respectively), who were also blinded to the type of oral contrast agent administered. Both readers independently assessed the diagnostic image quality in terms of the possibility to rule out a potential underlying pathology (two-point scale; yes or no). Further, the degree of luminal distension was evaluated on a three-point scale (0 = unsatisfactory distension, 1 = good distension, 2 = optimal distension).

### Radiation dose

The radiation dose was calculated for each CT exam using the computed tomography dose index (CTDI). Thoraco-abdominal examinations were scanned in one continuous scan, therefore the radiation dose of the thorax could not be excluded. Furthermore, a normalized CTDI was calculated dividing the CTDI by the body mass index (BMI).

### Statistical analysis

Statistical analysis was performed using JMP Pro 11 software (SAS Institute; U.S.A.) and GraphPad Prism 6 (GraphPad Software, Inc.; U.S.A.). Data are presented as mean with standard deviation. To test for potential differences in age and sex distribution between patients in the water and mannitol group the Kolmogorov-Smirnov test (after testing for a Gaussian and equal distribution using the Shapirow-Wilk normality test) was performed.

Quantitative and qualitative results as well as radiation doses were compared between the two study groups applying a Mann-Whitney test. In addition, a subgroup analysis was performed depending on scanner type. Further, interrater agreement was assessed using the intraclass correlation coefficient (ICC), applying the two-way mixed model [17]. ICC was interpreted as follows: a value less than 0.20 indicated poor agreement, a value of 0.21–0.40 fair agreement, a value of 0.41–0.60 moderate agreement, a value of 0.61–0.80 substantial agreement, and a value of 0.81–1.00 almost perfect agreement. For all measurements, a p-value less than 0.05 was considered indicative of a significant difference.

## Results

### Patients

In the group receiving water as an oral contrast agent, a thoraco-abdominal CT was acquired in 84 patients; in 8 patients only images of the abdomen were acquired. Respective numbers in the group receiving mannitol solution as an oral contrast agent were 86 and 2 patients. Patient characteristics as well as indications for staging CT in terms of underlying malignancy are presented in detail in Table 1. There were no significant differences between both study groups with regards to sex and age distribution (p>0.05).

**Table 1:** Characteristics of patients receiving either water or mannitol solution as neutral oral contrast agent.

	water group	mannitol group
No. of patients (total n = 180)	92	88
Sex		
Number of male patients	55	56
Number of female patients	37	32
Age (y)	59 (18–91)	63 (29–86)
Body mass index (kg/m^2^)	26.5 ± 5.4	25.7 ± 5.9
Primary tumor		
lip, oral cavity and pharynx	0	4
digestive organs	28	23
respiratory and intrathoracic organs	22	27
bone and articular cartilage	1	0
melanoma and other malignant neoplasms of skin	9	10
mesothelial and soft tissue	5	5
breast	3	3
female genital organs	3	1
male genital organs	4	5
urinary tract	7	2
thyroid and other endocrine glands	3	1
ill-defined, secondary and unspecified sites	0	2
lymphoid, hematopoietic and related tissue	7	5

Underlying malignancies were classified according to the World Health Organization (WHO)[18]. Age: mean with range in parenthesis. Body mass index (BMI): mean with SD.

### Quantitative assessment of intestinal distension

**Fig 4:**
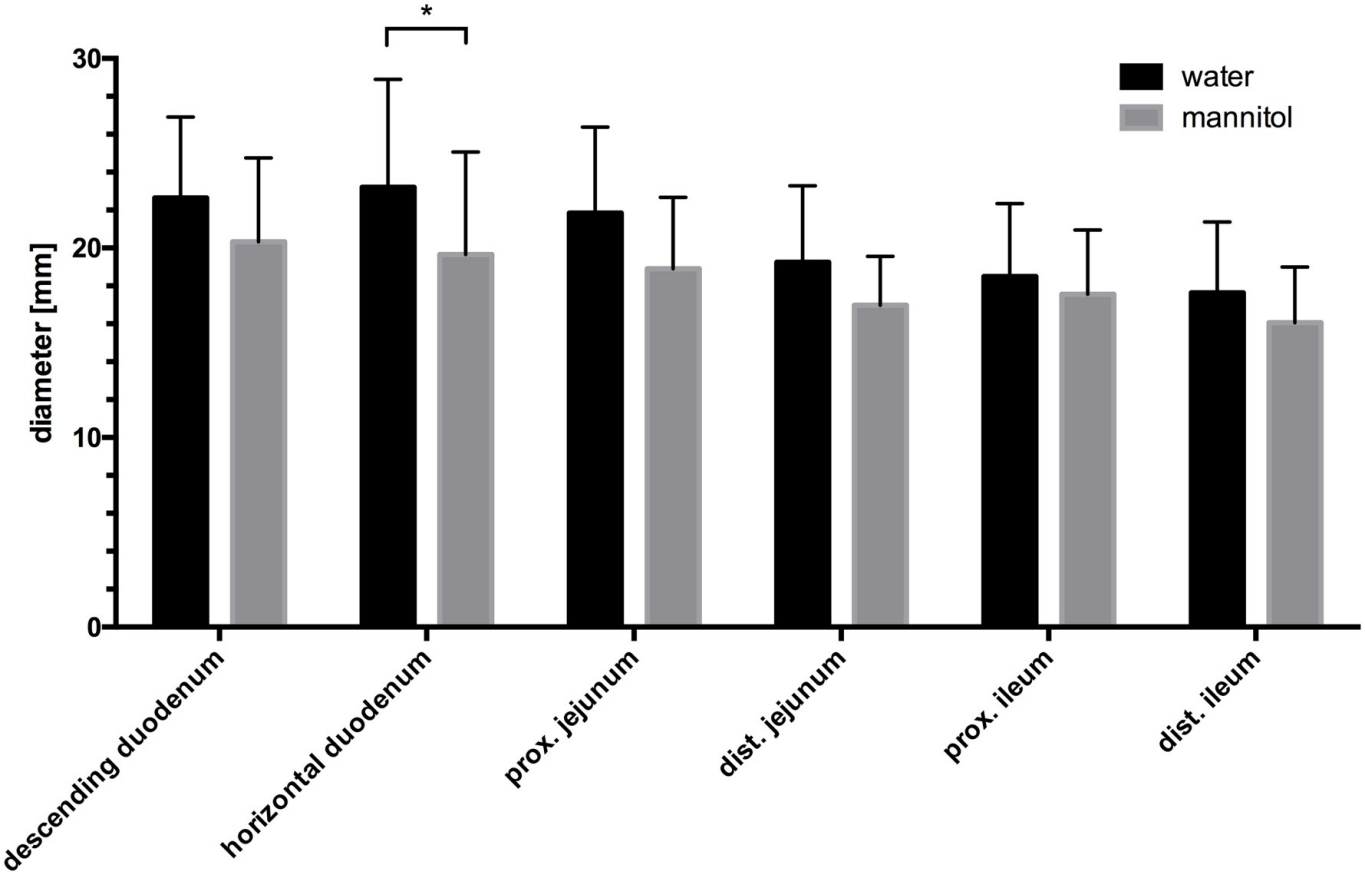
Mean diameter of each intestinal segment. Whiskers: upper confidence limit. Black columns: water group; grey columns: mannitol group. *: p< 0.05. Note the decrease of the diameter of the intestinal segments from proximal to distal.

Quantitatively, there was no statistically significant difference between patients receiving water and mannitol solution with regards to intestinal distension in almost all segments of the small intestine (p>0.05). Only in the horizontal duodenum intestinal distension was slightly better after water administration, with a mean diameter of 22.0 mm as compared to a mean diameter of 20.2 mm in the mannitol group (p = 0.019). In both groups, a decrease of the mean intestinal diameter was observed from oral to aboral (Fig 4). At subgroup analysis depending on scanner type, only distension in the horizontal duodenum was slightly better in examinations acquired on the 64-row CT using water as compared to mannitol (mean diameter of 22.6 mm vs. 20.6 mm; n = 71 and n = 61, respectively; p = 0.034). No significant difference between water and mannitol was observed in scans acquired at the 16-row CT (n = 21 and n = 27, respectively).

### Qualitative assessment of intestinal distension

Comparing the percentage of segments evaluated as diagnostic in terms of the possibility to rule out a potential underlying pathology, there was no statistically significant difference in the descending duodenum, horizontal duodenum or the proximal jejunum (both readers p>0.05; Table 2). Regarding the distal jejunum, both readers evaluated less segments as diagnostic in the water group as compared to the mannitol group (reader 1: 35% vs. 65%, p<0.0001; reader 2: 23% vs. 50%, p<0.001, respectively). Regarding the proximal and distal ileum, statistically significant more segments were rated as diagnostic after mannitol administration by reader 1 (water group vs. mannitol group: 43% vs. 67%, p = 0.002 and 56 vs. 75%; p = 0.009), whereas reader 2 found no significant difference between the two groups (64% vs. 77%, p = 0.054; and 48% vs. 52%, p = 0.553). Table 3 includes a subgroup analysis with further division of the water and mannitol group depending on the CT scanner used. A substantial bias introduced by the different CT could however not be found.

**Table 2:** Percentage of intestinal segments rated as diagnostic on a two-point scale in terms of the possibility to rule out a potential underlying pathology.

Segment	water group (n = 92)		mannitol group (n = 88)		p-value	
	R1 [%]	R2 [%]	R1 [%]	R2 [%]	R1	R2
descending duodenum	34	51	32	44	0.790	0.366
horizontal duodenum	18	33	18	30	0.961	0.659
proximal jejunum	8	18	8	20	0.934	0.740
distal jejunum	35	23	65	50	**<0.0001**	**<0.001**
proximal ileum	43	64	67	77	**0.002**	0.054
distal ileum	56	48	75	52	**0.009**	0.553
Mean percentage over all segments	32	40	44	46		

R1 = reader 1; R2 = reader 2; p-values: Mann-Whitney-test.

**Table 3:** Percentage of intestinal segments rated as diagnostic on a two-point scale in terms of the possibility to rule out a potential underlying pathology by CT-Scanner.

Segment	water group (n = 92)		mannitol group (n = 88)		p-value	
	R1 [%]	R2 [%]	R1 [%]	R2 [%]	R1	R2
**64-row CT**	n = 71		n = 61			
descending duodenum	35	51	36	44	0.921	0.463
horizontal duodenum	18	32	25	30	0.382	0.724
proximal jejunum	8	21	10	15	0.787	0.348
distal jejunum	34	24	61	43	**0.002**	**0.023**
proximal ileum	46	65	66	70	**0.029**	0.489
distal ileum	58	52	74	48	0.055	0.604
Mean percentage over all segments	33	41	45	42		
**16-row CT**	n = 21		n = 27			
descending duodenum	29	52	22	44	0.628	0.597
horizontal duodenum	19	33	4	30	0.091	0.796
proximal jejunum	5	10	4	33	0.881	0.056
distal jejunum	38	19	74	67	**0.014**	**0.001**
proximal ileum	33	62	70	93	**0.012**	**0.011**
distal ileum	52	33	78	63	0.069	**0.045**
Mean percentage over all segments	29	35	42	55		

R1 = reader 1; R2 = reader 2; p-values: Mann-Whitney-test.

**Fig 5:**
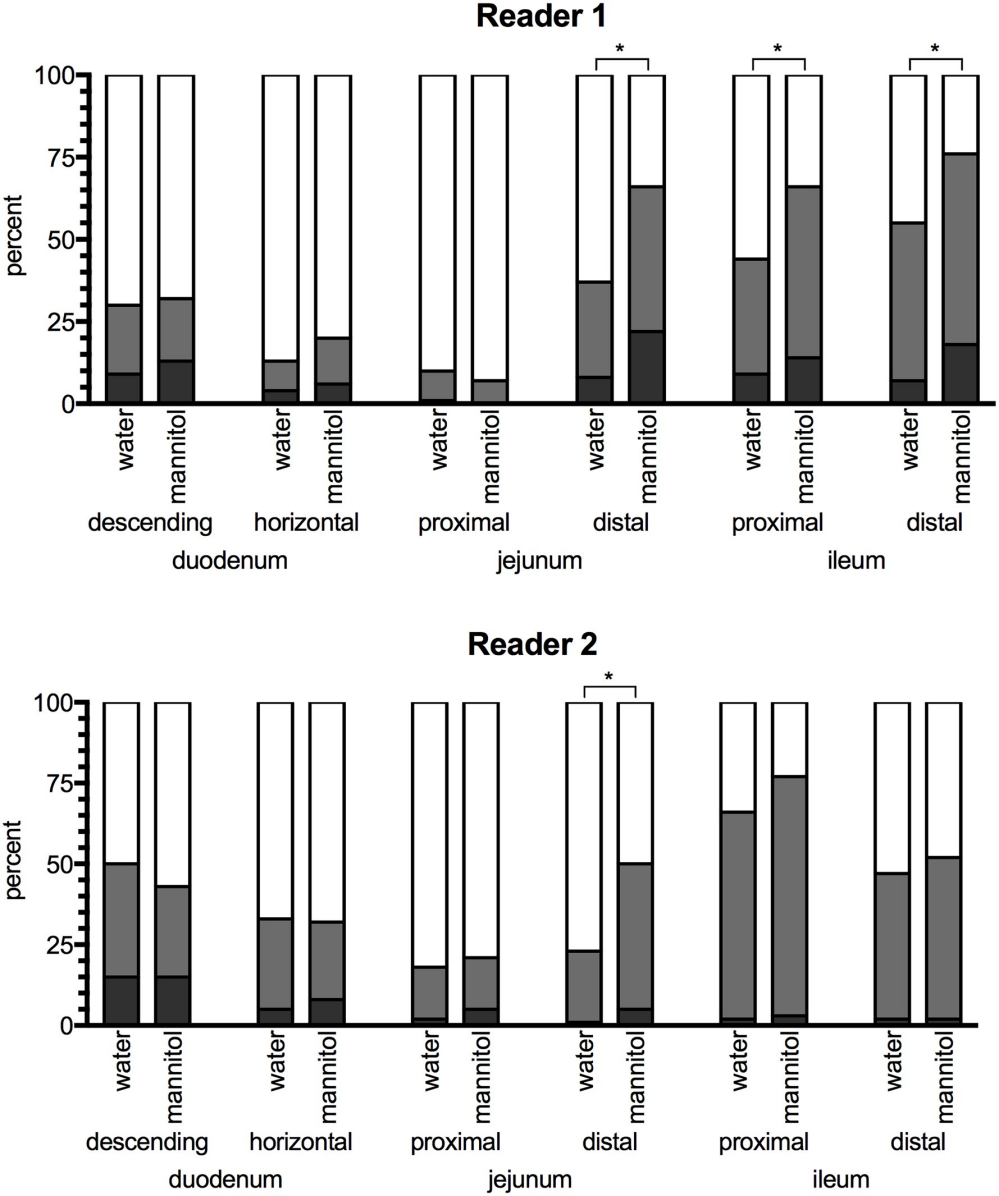
Rating of intestinal distension. Luminal distension was evaluated on a three-point-scale. Each bar, representing one segment, is divided according to the percentage of given ratings (white = unsatisfactory distension, grey = good distension, dark grey = optimal distension). *: p< 0.05.

Evaluating the degree of luminal distension as assessed on a three-point scale, no statistically significant differences between water and mannitol solution were observed in the three proximal diagnostically satisfactory intestinal segments (p>0.05 for both readers and all segments). However, both readers rated intestinal distension of the distal jejunum statistically significant better after administration of mannitol solution (both readers p<0.0001). In the two ileal segments, significantly better distension was observed only for reader 1 (p = 0.004 and 0.0001), whereas reader 2 rated both segments comparable (p = 0.098 and 0.471) (Fig 5). Overall, less than half of all intestinal segments were rated as diagnostic satisfactory (reader 1: water group 32%, mannitol group 44%; reader 2: water group 40%, mannitol group 46%).

**Fig 6:**
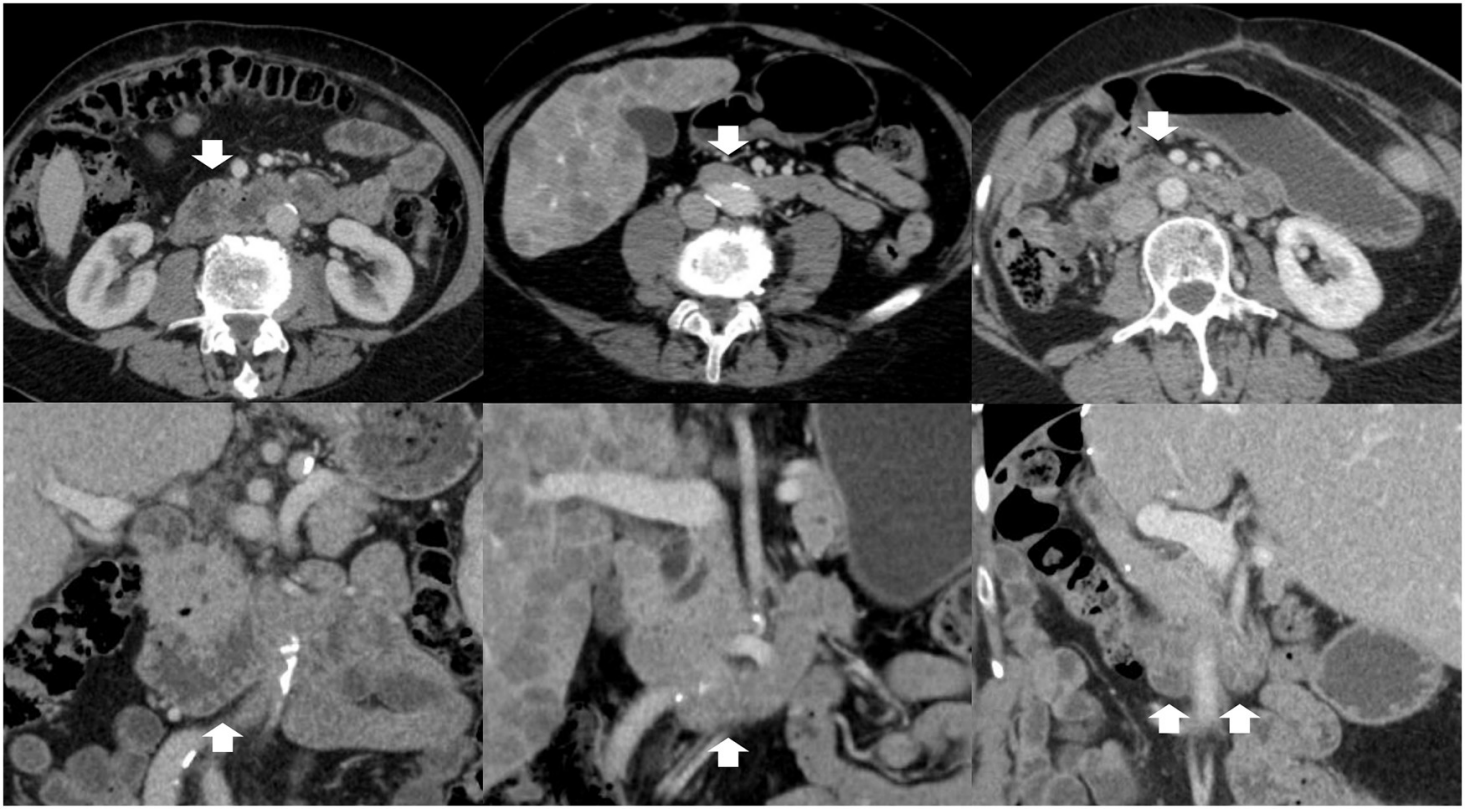
Interrater agreement horizontal duodenum. Exemplary slices of three patients (columns) in transverse (first row) and coronal plane (second row). First column: both readers rated the horizontal duodenum as diagnostic and degree of luminal distension as good; second column: both readers rated the horizontal duodenum as not diagnostic and degree of luminal distension as unsatisfactory. Third column; R1 rated the horizontal duodenum as diagnostic and degree of luminal distension as good while R2 did not.

Interrater agreement for assessment of intestinal distension in individual segments after water and mannitol administration was moderate, with a mean ICC of 0.52 and 0.49, respectively. Interrater agreement for individual intestinal segments dependent on the type of oral contrast agent is presented in detail in Table 4. Exemplary images of agreement and disagreement of both readers is presented in Fig 6.

**Table 4:** Intraclass coefficient for assessment of interrater agreement.

Segment	water group (n = 92)	mannitol group (n = 88)
	interrater agreement	interrater agreement
descending duodenum	0.563 (0.332–0.713)	0.548 (0.314–0.703)
horizontal duodenum	0.680 (0.479–0.799)	0.652 (0.469–0.771)
proximal jejunum	0.623 (0.432–0.750)	0.059 (-0.379–0.367)
distal jejunum	0.348 (0.035–0.562)	0.516 (0.242–0.688)
proximal ileum	0.402 (0.106–0.601)	0.638 (0.447–0.763)
distal ileum	0.475 (0.211–0.651)	0.531 (0.179–0.720)

Upper and lower confidence limits in parenthesis.

### Side effects

Side effects attributed to the oral contrast agent were observed only after mannitol administration, summing up to 29.5% (n = 26). Specifically, patients complained about diarrhea (n = 22; 25%), abdominal pain (n = 9; 10%) and nausea (n = 5; 6%, in one case with vomiting (1%). There were no adverse events attributed to the oral administration of water or to the intravenous contrast injection.

### Radiation dose

A comparison of the CTDI is shown in Table 5. Mean CTDI values were slightly higher in the water group compared to the mannitol group on both scanners without reaching statistical significance (p = 0.682). A subanalysis of each CT scanner alone showed statistically significant higher CTDI values for the water group compared to the mannitol group (p = 0.029) on the 16-row CT. However, after normalizing the CTDI values by a division with the patient’s body mass index in order to reduce the influence of patient’s weight and size, no significant difference could be found (p = 0.469).

**Table 5:** Radiation dose for both study groups based on the CT scanner used for imaging.

	16-row CT			64-row CT			16- and 64-row CT		
	water group (n = 21)	mannitol group (n = 27)	p	water group (n = 71)	mannitol group (n = 61)	p	water group (n = 92)	mannitol group (n = 88)	p
**BMI** [kg/m^2^]	27.1 (24.4–29.7)	24.2 (21.7–26.7)	0.112	26.4 (25.1–27.6)	26.4 (24.9–27.8)	0.900	26.5 (24.4–27.6)	25.7 (24.4–27.0)	0.330
**CTDI**									
absolute [mGy]	14.7 (13.6–15.9)	12.9 (11.6–14.1)	**0.048**	11.4 (10.4–12.4)	11.5 (10.0–12.9)	0.553	12.2 (11.3–13.0)	11.9 (10.8–13.0)	0.494
normalized [mGy*m^2^*kg^-1^]	0.54 (0.52–0.57)	0.63 (0.38–0.90)	0.344	0.43 (0.40–0.45)	0.42 (0.38–0.46)	0.478	0.46 (0.43–0.48)	0.49 (0.41–0.57)	0.942

Mean Body mass index (BMI) of the subgroups. Computed tomography dose index (CTDI) as absolute values and normalized (CTDI divided by BMI). Data is presented as mean with upper and lower confidence intervals in parenthesis.

## Discussion

In this prospective study we evaluated water and mannitol solution as neutral oral contrast agents for assessment of the small bowel in patients undergoing (thoraco-) abdominal staging CT. Even though individual distal segments were rated better at qualitative assessment using mannitol solution, this trend could not be verified quantitatively. Moreover, mannitol administration was associated with side effects in 29.5% of examinations, while patients receiving water reported no side effects. Radiation dose as assessed by CTDI was slightly lower after mannitol solution without reaching statistical significance.

Similar to our results, in a study comparing different administration techniques for CT enterography and enteroclysis, Paparo et al observed a declining intestinal distension from proximal to distal small bowel (jejunum to terminal ileum) using up to 2 liters of a neutral contrast agent administered orally or via a naso-jejunal tube. The reported intestinal distension of the ileal segments is in the range of our measurements, even though patients in our study received only 1 liter of an oral contrast agent. Interestingly, in their study a lower distension of the jejunum (mean 13 mm; range 10–25 mm) was observed as compared with the ileum (mean 17 mm; range 10–21 mm) [9]. Applying the contrast agent via a naso-jejunal tube (CT enteroclysis) improved distension of the jejunum (mean 27 mm; range 17–32 mm) only. Even though in our present study the distension of the jejunum was not as low as reported by Paparo, qualitative ratings were worse compared to the other intestinal segments. Hence, as Paparo pointed out, if the focus of the examination is a possible pathology in the jejunum, e.g. in patients with celiac disease, this might selectively affect this diagnostic accuracy. In such cases other techniques for intestinal distension (e.g. CT enteroclysis) should be considered. However, for patients undergoing (thoraco-) abdominal staging CT, the distension of jejunal loops achieved in the context presented in this study was acceptable.

Subjective ratings of intestinal distension and diagnostic quality were carried out in this study, reflecting how confident the two readers were in ruling out a possible underlying pathology. Regarding the two duodenal segments, our readers did not find significant differences between the water and mannitol group. The interrater agreement of the ratings was moderate to strong. Lowest percentages in terms of diagnostic quality were found for the proximal jejunum, also reflected by a high percentage of segments rated as unsatisfied distended and only poor to moderate agreement. A low distension might explain these conflicting results. The same may be true for the distal segments, in that a decreasing intestinal distension impedes bowel assessment.

This study focused on neutral oral contrast agents. A comparison with positive oral contrast agents was not carried out. In a recent study, Kammerer et al stated that for the majority of clinical indications neutral enteric contrast in abdominal CT imaging offers the most advantageous combination of bowel delineation, pathology detection and diagnostic reliability [19]. Other studies stated that water provides a sufficient enteric contrast for imaging specific gastrointestinal pathologies, e.g. chronic intestinal inflammation [2,7,20–23]. The use of positive oral contrast agents may be limited to distinct clinical questions such as pathologies with a lower density, e.g. fistulas or anastomotic insufficiency [19].

As known from the use of positive oral contrast agents, patients more often accept drinking the full amount of necessary neutral oral contrast agents without any additives [19]. In contrast to the mannitol group no patient in the water group reported any side effects (no diarrhea, nausea, vomiting or abdominal pain). Moreover, drinking water is advantageous in terms of hydration after i.v. contrast application [24]. Furthermore, from an economical point of view, using water without mannitol as an oral contrast agent results in slightly lower costs (difference around € 4.00 per patient).

Our study had several limitations. The same time schedule was applied for administration of the negative oral contrast agents 45 minutes prior to CT examination, although water may have a faster transit time compared to mannitol. Thus the clinical workflow established in our institute has not to be changed, which might introduce unnecessary errors. Evaluation of different time schemes was not carried out. For quantitative measurements the total diameter as the intestine was regarded as the luminal diameter, as the intestinal wall was not always distinguishable from the lumen. Thus intestinal wall thickening (caused by e.g. contractions or wall edema) may have distorted the measurements. We tried to compensate for this using with the additional qualitative assessment. Qualitative assessment was carried out by two relatively inexperienced radiology residents, which in part might be a possible explanation for discrepancies regarding the evaluation of intestinal distension. The chosen three-point scale for assessment of the distension might have lowered reproducibility as well. Our readers were asked how confident they were in terms of ruling out a possible underlying pathology without any comparison to a reference standard (e.g. pathologic diagnosis).

In conclusion, we accept the null hypothesis that there is no difference between orally administered water and mannitol solution for evaluation of the small bowel at abdominal staging CT in clinical routine only for the quantitative, but not for the qualitative analysis. Furthermore, looking more differentiated at the overall performance, water has advantages in terms of patient comfort, side effects and costs, and can therefore be regarded as noninferior to mannitol in this specific patient group.
